# Does Foraging Performance Change with Age in Female Little Penguins (*Eudyptula minor*)?

**DOI:** 10.1371/journal.pone.0016098

**Published:** 2011-01-25

**Authors:** Ilka Zimmer, Yan Ropert-Coudert, Akiko Kato, Andre Ancel, Andre Chiaradia

**Affiliations:** 1 Université de Strasbourg, IPHC, Strasbourg, France; 2 CNRS, UMR7178, Strasbourg, France; 3 National Institute of Polar Research, Tokyo, Japan; 4 Research Department, Phillip Island Nature Park, Cowes, Victoria, Australia; Institute of Ecology, Germany

## Abstract

Age-related changes in breeding performance are likely to be mediated through changes in parental foraging performance. We investigated the relationship of foraging performance with age in female little penguins at Phillip Island, Australia, during the guard phase of the 2005 breeding season. Foraging parameters were recorded with accelerometers for birds grouped into three age-classes: (1) young, (2) middle age and (3) old females. We found the diving behaviour of middle-aged birds differed from young and old birds. The dive duration of middle age females was shorter than that of young and old birds while their dive effort (measure for dive and post-dive duration relation) was lower than that of young ones, suggesting middle-aged birds were in better physical condition than other ones. There was no difference in prey pursuit frequency or duration between age classes, but in the hunting tactic. Females pursued more prey around and after reaching the maximum depth of dives the more experienced they were (old > middle age > young), an energy saving hunting tactic by probably taking advantage of up-thrust momentum. We suggest middle age penguins forage better than young or old ones because good physical condition and foraging experience could act simultaneously.

## Introduction

In vertebrates, survival and reproductive performance generally increase with age [1], [2], [3]. However, for long-lived species, this increase is often followed by stabilization in survival and reproductive performance at middle age, then a decline in old age, e.g. [1], [3], [4], [5], [6], [7]. This decline, or senescence, involves a loss of physiological functions and is accompanied by decreasing fertility and increased risks of mortality with advancing age [5].

Age-related changes in breeding performance are known to be the result of experience accumulated over time in several aspects of breeding in birds i.e. laying date, nest site selection, foraging [8]. The improvement in breeding success is likely to be mediated through improvement in individual foraging ability, which would correspond to an enhanced capacity to provide for the offspring [9]. Foraging involves a hierarchical process of decision-making [10], including choosing appropriate habitat/patch; searching for or/and recognizing suitable prey; and capturing them. At each of these stages, young individuals may experience deficiencies, i.e. less or lower energetic food supply to their offspring. Improved foraging performance at older ages has been explained by an increase in foraging efficiency with experience in birds [11], related to improvements in foraging ability [12], diet choice [13] and access to better foraging territories [14].

Seabirds usually have long life spans and slow aging rates in relation to their body size [15], which makes them optimal models for studying aging processes. Seabird studies of the influence of age on foraging performance are challenging because: 1) long-term demographic datasets are scarce [16] and, as in all age-related studies, it is difficult to have a various sample size in age up to old age and 2) at-sea behaviour can only be recorded by remote sensing technologies compared to studies on age-related breeding performance on land. Hence only few studies have yet investigated the influence of age on seabird foraging performance in the wild [11], [17].

In the present study we used an avian long-lived marine top predator, the little penguin (*Eudyptula minor*) as a model to examine age-related foraging behaviour since age-related differences in breeding performance are well documented [7]. We investigated whether age affects penguin foraging performance by examining diving and hunting behaviour in relation to age.

## Methods

### Ethics Statement

Field work protocol was approved by the ethics committee of the Phillip Island Nature Park with a research permit issued by the Department of Sustainability and Environment of Victoria, Australia.

### Fieldwork

This study was conducted on the little penguin breeding colony situated at Phillip Island Nature Parks (38°31′S, 145°09′E), Victoria, Australia. In order to reduce the variability of other influencing factors which may confound the effect of age on foraging performance, we investigated only breeding females at guard phase during one single season. During guard phase, breeding little penguins make one-day foraging trips since they alternate daily to attend small chicks of up to three weeks old [18]. Thus age-related differences in foraging performance would not be masked by foraging trip duration and sexual differences [19].

From November to December 2005 we equipped 19 breeding females with miniature accelerometers to monitor their foraging activity (M190-D2GT, Little Leonardo, Tokyo, Japan). The mean body weight of females was 1042±77 (SD) g. Depth was measured every second with an accuracy of ±1 m and a resolution of 0.05 m. Acceleration was measured along the longitudinal body axis and the dorso-ventral axis between 0 and ±30 m s^−2^ at 32 Hz. This sensor measured both dynamic acceleration (i.e., vibration) and static acceleration (i.e., gravity). The accelerometers were cylindrically shaped four-channel data loggers with domed heads (15×53 mm, 17 g) that accounted to 3.4% of the penguins' frontal area [20]. In stream-lined marine animals such as penguins it is the size of this frontal area which may influence the foraging behaviour [21], including little penguins [20]. Therefore, we followed recommendations to reduce possible logger influences in this study. Females were caught in their nest boxes and the accelerometer was attached on the lower back of the bird to reduce hydrodynamic drag [21] using waterproof tape preserving the integrity of the plumage [22], [23]. This method further allowed us to minimize the handling time, attaching and retrieving the logger in less than 5 min before birds were returned to their nest-boxes. After a single foraging trip, birds were recaptured in their nest boxes and the logger and tape were removed before birds were released. The bird's breeding activities were then monitored until chicks fledged or died. Penguins were marked with electronic transponders (Allflex, Australia Pty Ltd, Capalaba, Queensland, Australia, www.allflex.com.au) either before fledging or at their first sighting in this study area of the Phillip Island breeding colony to monitor penguins' arrivals and departures through an automated penguin-monitoring system installed on their natural path [24]. The transponders (23×3.8 mm), supplied in individually packed sterilized needles, were injected under the loose skin on the back of the penguin's neck and the wound was closed with surgical glue (Vetbond™, 3 M worldwide, www.3m.com) to prevent infection and transponder loss. Animal contact was kept to a minimum; the transponder injection taking less than 1 min. At Phillip Island, transponders have been used since 1994 and no ill effects have been reported, i.e. no tissue damage or transponder migration from injection site [25]. Field work protocol was approved by the Animal Experimentation Ethics Committee, Phillip Island Nature Park (PINP AEEC, number PINP AEEC 2.2004) with a research permit issued by the Department of Sustainability and Environment, Flora and Fauna (number 10003419) of Victoria, Australia. In accordance with recommendations by these committees, procedures were adopted to minimise the impact of sampling protocol. These include: i) only marked birds with known breeding status were targeted to avoid repeated sampling on the same individuals in the same breeding season. No individual bird were sampled more than once in a breeding season; ii) only females over 800 g; iii) Birds were sampled on the point of collection to reduce handling and retaining time; iv) Procedures were ceased immediately if serious signs of distress were detected which is indicated by abnormal breathing and over-dilated pupils during handling – we did not face with any such case in this study; v) Birds were released immediately after the logger was attached. Procedure took less than 5 minutes; vi) Information on each sampled bird were entered in a database to prevent re-sampling of the same bird.

All studied females were of known age. Their age ranged from three to fourteen years. Age could not be treated as continuous variable since the sample size per year was unequal and severely reduced in some years. We then treated age as categorical variable by grouping them in three age classes following [25]: class 1 for young birds (3 and 4 years, n = 7), class 2 for middle-aged birds (5 to 10 years, n = 7) and class 3 for old birds (11 to 14 years, n = 5). These three age groups coincide with the curvilinear relationship between age and breeding success [7]. By grouping the data, we could try to detect non-linear relationships which we would not be possible with continuous analysis due the limitations of our dataset.

### Dive analysis

The dive analysis was conducted on depth and acceleration data using IGOR Pro (Wavemetrics Inc., USA, 2008, Version 6.04). Data were corrected for surface drift and according to the resolution of the loggers only dives >1 m were considered for analysis. The software analyzed dives sequentially (dive by dive analysis), writing a number of parameters for each dive into an output file. These were: maximum dive depth (m), total dive and post-dive duration (s), descent, bottom and ascent duration (s), and the number and duration of dashes (s), as a proxy of the prey pursuit [26], [27].

**Figure 1:**
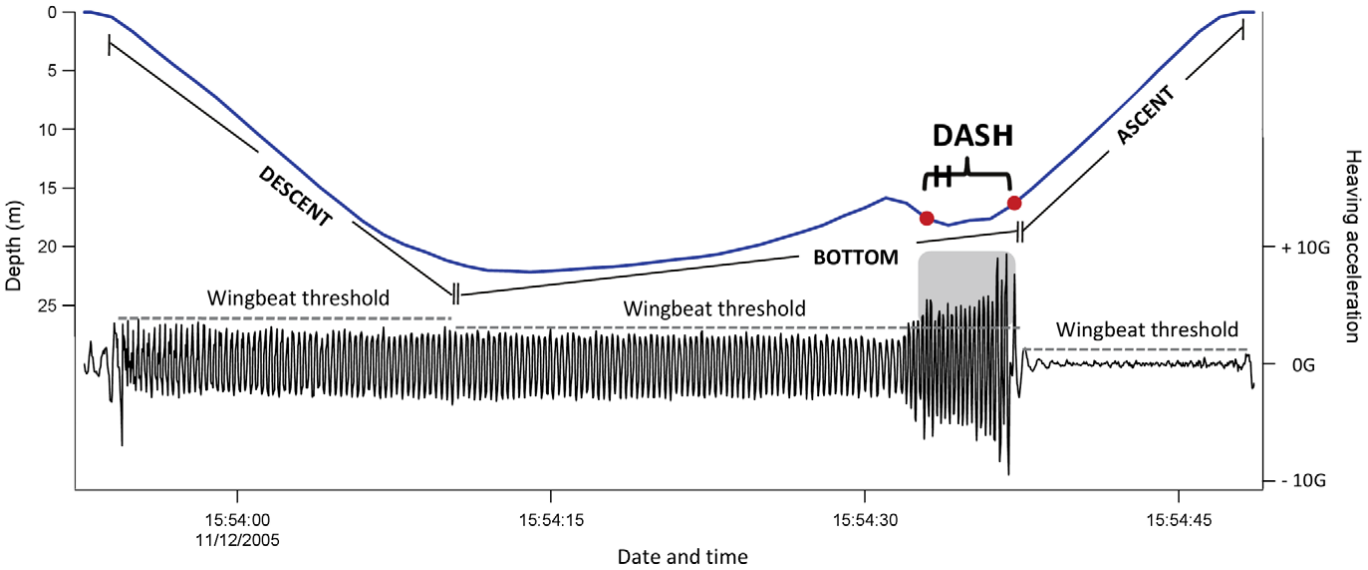
Example of a little penguin foraging dive and the heaving acceleration used to analyse wing beat amplitude and frequency. Upper thresholds were defined for the regular wing beat amplitude of the descent, bottom and ascent phase, being highest during descent and lowest during ascent. Prey pursuits (dashes) were considered when the wing beat amplitude was greater than the threshold of the respective dive phase for more than three consecutive wing beats as shown by the dash example.

A dive started and ended when birds dived from and returned to the water surface. The start and end of bottom phases were defined as the first and last time in a dive when the rate of change of depth became <0.25 m.s^−1^ [28]. The post-dive duration, representing the recovery time between dives at the surface, was considered when ≤100 s [29]. Flipper beats were apparent in the acceleration signals as an oscillating pattern being simultaneously present on both axes, with each propulsive stroke recorded on the heaving axis resulting in a forward acceleration recorded on the surging axis. The amplitude and frequency of each wing beat were analysed using the heaving acceleration signal (the most sensitive to undulation in the birds' body resulting from flipper beats). We found there was an upper amplitude threshold for regular diving wing beats per dive phase, being highest during descent and lowest during ascent (see Fig. 1). Wing beat characteristics differed between individuals so that the threshold was adjusted for the descent, bottom and ascent phase of each bird. Dashes were considered when the wing beat amplitude became greater than this defined threshold for more than three consecutive wing beats (Fig. 1). The first 8 m of descent phases were excluded from dash detection because birds stroke their wings hardly to overcome buoyancy in the beginning of a dive and do therefore not represent the regular wing beat pattern.

As a physical index to represent dive effort (DE), which considers the relationship between dive duration and post-dive duration, we divided the dive duration by the dive cycle (dive duration/dive duration + post-dive duration). Moreover, the acceleration records in this study provided the dash duration and allowed us to derive an efficiency index to indicate the effort spent in pursuing prey, here labelled as hunting efficiency (HE = sum of dash duration/dive cycle duration). We note that, even though the dash event is not a direct and quantative measure of prey ingestion, it remains a qualitive measure for prey encounter and pursuit. Once appropriate prey is found the chances to catch it are expected be high [30] and hence the capacity to pursue prey would be more sensitive to age related changes than the catch itself. In penguins a quantitative measure for prey ingestion remains still challenging. DE and HE are expressed as non-dimensional units.

In order to investigate where prey pursuit occurred within dives we examined dash frequency at any given depth. We compared the dash frequency among three categories (A, B, C) of depth percentage from the maximum dive depth, to indicate where in a single dive most prey were pursued: either (A) at depths shallower than 80% of maximum depth before reaching the maximum dive depth, coinciding with descent (i.e. from 0% to 80%), (B) in the deepest part of a dive around the bottom (i.e. from 80% to −80%), or (C) at depths shallower than −80% of maximum dive depth after penguins had reached the maximum dive depth, coinciding with ascent (i.e. from −80% to 0%). To avoid the high influence of upthrust in the upper part of the water column [31] we only included dives >8 m.

We moreover investigated the dash orientation, i.e. if penguins swam predominantly downward or upward when pursuing prey. This was calculated by subtracting the end depth of each single dash with the start depth of the dash (depth values being positive), so that negative results indicated upward movement.

In addition to the above parameters, which are based on a dive-by-dive analysis, we investigated two foraging parameters which provide information on the whole foraging trip (foraging trip based). These were the total time spent underwater, calculated as the sum of dive durations, and the total dash number, calculated as the sum of dashes per foraging trip and per penguin.

### Statistics

To compare the diving behaviour between the three age classes, we used General Linear Mixed Models to avoid pseudo replication [32]. Individuals were considered as random factors while the age-class was a fixed factor. If data were not normally distributed they were log10 transformed. Since the maximum depth influences all other dive parameters [33], [34], we included maximum depth as a covariate into the model and calculated least square means per bird so as to compare among age classes independent on depth. We provide the denominator degrees of freedom. The numerator degree of freedom is equal to two for all diving parameters. For the two foraging trip based parameters, total time spent underwater and total dash number, the effect of age classes have been tested by General Linear Models as the each individual has only one value. Generalized Linear Models (GLM) were used with binominal distribution and post-hoc Tukey's test to test the difference in the proportion of dashes which occurred in the different parts of the dive among age classes. To test the differences among age classes in orientation of the dashes, we used a paired t-test and post-hoc ANOVA.

Analyses were conducted using JMP 8 (SAS Institute Inc.) and R 2.10.1 (R Development Core Team). Results are expressed as means ± SE and significance level was set at α = 0.05.

## Results

All 19 loggers were recovered and full datasets were downloaded successfully from all devices. The mean dive depth did not differ between age classes (Table 1). Middle age penguins showed differences in their foraging performance compared to young and old birds. These differences were related to the dive duration parameters and the within-dive orientation of dashes.

**Table 1:** General Linear Mixed Model and Tukey's HSD test results for age class comparison of diving parameters.

	Young (N = 7)		Middle (N = 7)		Old (N = 5)		*F*	*dfd*	*P*
	mean	SE	mean	SE	mean	SE			
Mean dive depth (m)	7.72_a_	1.46	8.45_a_	1.46	10.57_a_	1.73	0.83	15.8	0.456
Mean dive duration (s)*	10.32_a_	1.93	7.73_b_	1.03	10.83_a_	0.90	10.66*	16.1	**0.001**
Mean descent duration (s)*	3.08_a_	0.57	2.41_b_	0.18	3.10_a_	0.17	8.00*	16.1	**0.004**
Mean bottom duration (s)*	5.19_ab_	1.05	4.46_b_	1.06	5.96_a_	0.60	3.45*	15.8	0.057
Mean ascent duration (s)*	3.07_a_	0.40	2.43_b_	0.20	3.12_a_	0.12	12.47*	16.1	**0.001**
Mean post-dive duration (s)*	13.82_a_	2.84	17.04_a_	4.83	15.98_a_	2.44	1.25*	16.0	0.313
Total time spent underwater (h)	5.07_a_	0.50	4.78_a_	0.50	6.53_a_	0.59	2.83	16	0.089
Mean dive effort*	0.33_a_	0.08	0.24_b_	0.07	0.32_ab_	0.05	3.98*	16.0	**0.040**
Mean dash number	1.63_a_	0.07	1.52_a_	0.07	1.56_a_	0.09	0.59	16.6	0.567
Total dash number	322_a_	62	281_a_	62	185_a_	73	1.05	16	0.373
Mean dash sum duration (s)*	2.23_a_	0.72	2.07_a_	0.56	2.71_a_	0.74	1.24*	15.9	0.316
Mean hunting efficiency*	0.06_a_	0.02	0.05_a_	0.01	0.07_a_	0.02	1.13*	15.4	0.349

Least-square means were used for comparison. Denominator degrees of freedom (dfd) are provided. Numerator degree of freedom equal 2 for all parameters. Significant results are in bold. The results of post-hoc Tukey's HSD test are shown by the subscript letters: same letter showed no significant differences.

*Data were log10 transformed for the statistical analysis.

Middle age penguins made shorter dives than young or old penguins (Table 1). This was mainly due to middle age penguins having shorter descent and ascent phases, while the bottom duration showed a similar trend but was not significantly different between the three classes (Table 1). Post-dive duration was not significantly different between age classes (Table 1). The total time spent underwater during a foraging trip did not differ among age classes (Table 1).

Middle age penguins showed lower dive effort (DE) than young birds (Table 1). We compared mean dash number, total dash number and mean dash sum duration between age classes, but there were no significant differences (Table 1). In addition, hunting efficiency (HE) did not differ between age classes (Table 1).

**Figure 2:**
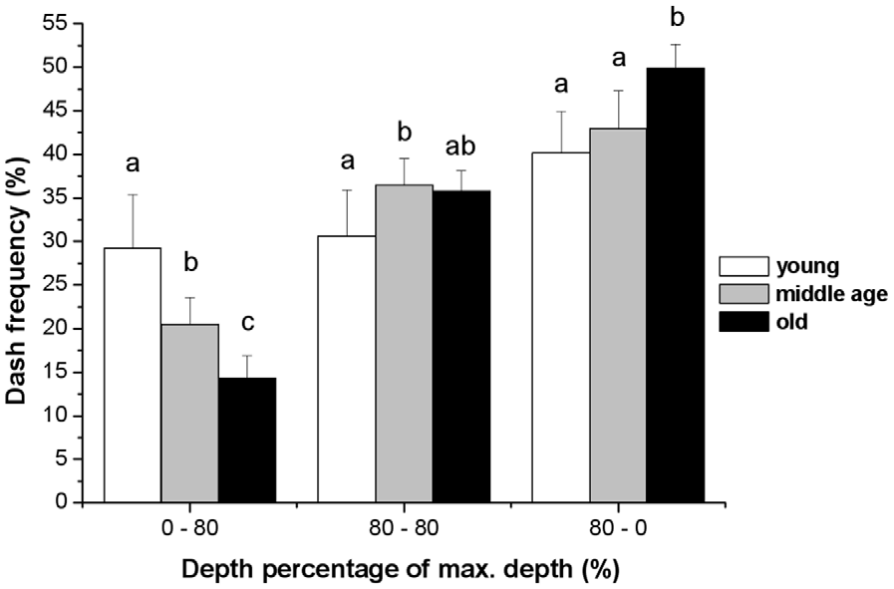
Prey pursuit (dash) frequency (%) in relation to the depth percentage of the max. dive depth (%) in three categories (see methods): (A) 0–80%, (B) 80–80% and (C) 80–0% for young, middle age and old little penguins. The results of post-hoc Tukey's test are shown by the bold letters: same letter showed no significant differences between age classes.

The proportion of dashes, before (0–80%), around (80–80%) or after (80–0%) reaching the deepest point of dives, were different among age classes (0–80%, LRT = 51.5, P<0.001, 80–80%, LRT = 10.8, P<0.01, 80–0%, LRT = 14.0, P<0.001, df = 2, Fig. 2). In 0–80% depth, young birds pursued prey more often than middle age (z = −4.7, P<0.001) and old birds (z = −6.7, P<0.001), and middle-aged birds pursued more than old birds (z = −2.8, P<0.05). In 80–80% depth, young birds pursued prey less often than middle-aged birds (z = 3.2, P<0.01) and there was no difference between young and old birds (z = 2.1, P = 0.08) or between middle age and old birds (z = −0.73, P = 0.74). In 80–0% depth, old birds pursued prey more often than young (z = 3.6, P<0.01) and middle-aged birds (z = 2.9, P<0.05); there was no difference between young and middle-aged birds (z = 0.94, P = 0.61).

We moreover found that all penguins performed more upward-orientated dashes than downward-oriented ones (paired t-test, *t*_18_ = 2.9, *P*<0.01, Table 2), without differences between age-classes (post-hoc ANOVA, *F*_2, 16_ = 0.26, *P* = 0.77).

**Table 2:** Dash end depth frequency (%) in relation to the hunting direction: downward and upward orientation for young, middle age and old little penguins.

	Downward	Upward
Young (N = 7)	41.0	59.0
Middle age (N = 7)	41.0	59.0
Old (N = 5)	42.7	57.3

No siginificant differences were found between age classes.

## Discussion

Our results highlighted differences in the foraging activity of little penguin females according to their age. In several key variables used to measure diving behaviour, middle-aged females have showed better foraging performance than young and old penguins. Middle-aged females made shorter duration dives, getting to the bottom of the dive faster while reaching similar depths with less effort than the other age groups. These trend in age performance coincided with the curvilinear trend observed for breeding performance and group behaviour differences related to age on little penguins [7], [25]. Since breeding success is closely related to foraging efficiency [35], [36], [37], we suggest that middle age females could be better at bringing food to their chicks compared with other age classes.

We grouped our data in three age classes rather than continuous data in the analysis due to the limitations in our dataset. Nevertheless we could detect age-related change in the dive parameters among the groups. This is an advantage compared with most foraging studies which compare only two age classes, but see [13], like a recent study [17], which could therefore only detect the initial increase in foraging ability from young to adult common terns *Sterna hirundo* while a change with age in old birds might have been overlooked.

If young breeders are less proficient foragers than experienced ones, the age difference may be expressed in several ways. (1) Young birds may spend more time foraging, like experienced terns that cover their feeding areas more quickly than inexperienced ones [38], [39]. Although we did not find an age-related difference in the total time spent underwater, we found differences in the organisation of the different phases of the dives. While our guarding penguins were constrained to forage within only one-day period, we could still reveal a trend as young inexperienced and older females had longer dive durations than middle age ones. (2) Young birds might also use different feeding spots or simply feed on different sizes or types of food, smaller or of less energetic value [17], [38], [39]. Several avian studies have reported an improvement in foraging efficiency in terms of rate of energy gain [11]. We examined the relation of age and foraging performance by using high-resolution data, which allowed us to investigate if there were differences in prey pursuit patterns at a fine scale. Hence we expected young females to have lower hunting efficiency and prey pursuit frequency or duration because these are the most obvious parameters to indicate foraging efficiency, but we did not find any significant differences. Since we did not know the chick provisioning rate, this does not necessarily mean that middle age and old birds were more or less successful capturing prey [11]. However, we found a marked difference in the tactic of prey pursuit in the hunting pattern within dives among the three age classes. Prey pursuit increases across the age so more experienced birds (old > middle age > young) could take advantage of up-thrust momentum, expanding air in respiratory system [40] which facilitates acceleration to pursue prey. This prey pursuit pattern suggests an energy saving hunting skill [41] probably learned through age and foraging experience [42], [43].

The similar response in dive durations of young and old females in our study can result from different factors. While young females may not have fully developed their foraging skills, old females are probably affected by senescence and their progressive decline in physical condition [44]. Thus, we can suspect that middle age females combine well-developed foraging skills with good physical condition since they did not rely on longer dive durations to search or pursuit prey while taking advantage of the same post-dive duration for recovery at the surface, shown by their lower dive effort. Other factors could affect dive-pause ratio of diving seabirds, i.e. when prey density or the probability of losing contact with ephemeral prey is high [45]. But since old females dived longer with the same prey pursuit rate as other age classes (there was no difference in the number or duration of prey pursuit between age classes); we suggest that older females have lower prey pursuit ability despite their use of energy saving hunting tactics. Senescence is the ‘persistent decline in age-specific fitness components of an organism due to internal physiological deterioration’ [44], which is genetically determined [46]. One hypothesis about responsible intrinsic factors for aging processes is the oxidative theory of aging [47]. Cumulative damage on cell constituents can progressively reduce resistance to oxidative stress, leading to a gradual aging process. In greater flamingos this theory has been supported by a curvilinear relationship with middle-aged birds showing a greater resistance to oxidative stress than young and old ones [48]. Decline in hunting ability due to physiological deterioration at old age was found in wolves [49] and aboriginal human hunters [50], also supporting a curvilinear relation with age. Physiological deterioration at old age may be, however, balanced by increasing foraging experience until a certain age limit, which may well be related to our birds in the transition stage between middle age and old birds. Since it is often difficult to study the oldest individuals [51] in wild populations, the nature of such limits is not fully understood yet and remains a challenge to future studies.

Our 2005 study season was a highly productive season, as indicated by high penguin breeding success [26], and we may expect age-related differences to be more distinct when environmental conditions are poorer, i.e. lower prey availability [52]. Also, our cross-sectional foraging study should be regarded as a snapshot within one breeding season. In order to confirm our hypothesis future studies should investigate age-related foraging performances over the complete breeding season, as well as across years, and ideally at a longitudinal scale.

Behavioural studies which aim to extrapolate their findings on the population level require the consideration of individual age differences. The behaviour of a whole population is usually assumed to be the sum of individual behaviour, disregarding the age structure of the population. By investigating the foraging performance in relation to age, future studies could make more accurate predictions on population trends. In this case, a population with a high proportion of middle age individuals would be expected to be more successful in foraging, and therefore chick provisioning, than a population dominated by young and old individuals. Thus, a population dominated by middle age individuals would better contribute to the next generation. This contribution would be especially relevant in years of low resource availability and probably influence the response of a population to environmental changes, which should be considered in conservation and management of populations.

## References

[1] Clutton-Brock TH (1988). Reproductive success: studies of individual variation in contrasting breeding seasons..

[2] Newton I (1989). Lifetime reproduction in birds..

[3] Forslund P, Pärt T (1995). Age and reproduction in birds: hypotheses and tests.. Trends Ecol Evol.

[4] Weimerskirch H (1992). Reproductive effort in long-lived birds: age-specific patterns of condition, reproduction and survival in the wandering albatross.. Oikos.

[5] Kirkwood TBL, Austad SN (2000). Why do we age?. Nature.

[6] Reid JM, Bignal EM, Bignal S, McCracken DI, Monaghan P (2003). Age-specific reproductive performance in red-billed choughs Pyrrhocorax: patterns and processes in a natural population.. J Anim Ecol.

[7] Nisbet I, Dann P (2009). Reproductive performance of little penguins *Eudyptula minor* in relation to year, age, pair-bond duration, breeding date and individual quality.. J Avian Biol.

[8] Pärt T (1995). Does breeding experience explain increased reproductive success with age?. Proc R Soc B.

[9] Stearns SC (1992). The evolution of life histories..

[10] Wunderle JM (1991). Age-specific foraging proficiency in birds.. Curr Ornithol.

[11] Daunt F, Wanless S, Harris MP, Money L, Monaghan P (2007). Older and wiser, improvements in breeding success are linked to better foraging performance in European shags.. Func Ecol.

[12] Daunt F, Wanless S, Harris MP, Monaghan P (1999). Experimental evidence that age-specific reproductive success is independent of environmental effects.. Proc R Soc B.

[13] Rutz C, Whittingham MJ, Newton I (2006). Age-dependent diet choice in an avian top predator.. Proc R Soc B.

[14] Pärt T (2001). The effects of territory quality on age-dependent reproductive performance in the northern wheatear, Oenanthe oenanthe.. Anim Behav.

[15] Holmes DJ, Austad SN (1995). The evolution of avian senescence patterns: implications for understanding primary aging processes.. Am Zool.

[16] Wooller RD, Bradley JS, Croxall JP (1992). Long-term population studies of seabirds.. Trends Ecol Evol.

[17] Limmer B, Becker PH (2009). Improvement in chick provisioning with parental experience in a seabird.. Anim Behav.

[18] Chiaradia A, Nisbet I (2006). Plasticity in parental provisioning and chick growth in Little Penguins *Eudyptula minor* in years of high and low breeding success.. Ardea.

[19] Bethge P, Nicol S, Culik BM, Wilson RP (1997). Diving behaviour and energetics in breeding little penguins (*Eudyptula minor*).. J Zool.

[20] Ropert-Coudert Y, Knott N, Chiaradia A, Kato A (2007). How do different data logger sizes and attachment positions affect the diving behaviour of little penguins?. Deep-Sea Res II.

[21] Bannasch R, Wilson RP, Culik B (1994). Hydrodynamic aspects of design and attachment of a back-mounted device in penguins.. J Exp Biol.

[22] Wilson RP, Wilson M-P (1989). Tape: a package attachment technique for penguins.. Wildl Soc Bull.

[23] Wilson RP, Pütz K, Peters G, Culik B, Scolaro JA (1997). Long-term attachment of transmitting and recording devices to penguins and other seabirds.. Wildl Soc Bull.

[24] Chiaradia A, Kerry K (1999). Daily nest attendance and breeding performance in the Little penguin *Eudyptula minor* at Phillip Island, Australia.. Mar Ornithol.

[25] Daniel TA, Chiaradia A, Logan M, Quinn GP, Reina RD (2007). Synchronized group association in little penguins, Eudyptula minor.. Anim Behav.

[26] Ropert-Coudert Y, Kato A, Chiaradia A (2009). Impact of small-scale environmental perturbations on local marine food resources: a case study of a predator, the little penguin.. Proc R Soc B.

[27] Ropert-Coudert Y, Kato A, Wilson RP, Cannell B (2006). Foraging strategies and prey encounter rate of free-ranging little penguins.. Mar Biol.

[28] Kato A, Ropert-Coudert Y, Chiaradia A (2008). Regulation of trip duration by an inshore forager, the little penguin (*Eudyptula minor*), during incubation.. Auk.

[29] Chiaradia A, Ropert-Coudert Y, Kato A, Mattern T, Yorke J (2007). Diving behaviour of Little Penguins from four colonies across their whole distribution range: bathymetry affecting diving effort and fledging success.. Mar Biol.

[30] Simeone A, Wilson RP (2003). In depth studies of Magellanic penguin (*Spheniscus magellanicus*) foraging: can we estimate prey consumption by perturbations in the dive profile?. Mar Biol.

[31] Wilson RP, Hustler K, Ryan PG, Burger AE, Noldeke EC (1992). Diving birds in cold water - do Archimedes and Boyle determine energetic costs?. American Naturalist.

[32] Zuur AF, Ieno EN, Walker NJ, Saveliev AA, Smith GM (2009). Mixed effects models and extensions in ecology with R..

[33] Wilson RP, Bost CA, Pütz K, Charrassin JB, Culik BM (1997). Southern rockhopper penguin *Eudyptes chrysocome chrysocome* foraging at Possession Island.. Polar Biol.

[34] Cherel Y, Tremblay Y, Guinard E, Georges JY (1999). Diving behaviour of female northern rockhopper penguins, *Eudyptes chrysocome moseleyi*, during the brooding period at Amsterdam Island (Southern Indian Ocean).. Mar Biol.

[35] Morrison ML, Ralph CJ, Verner J, Jehl JRJ (1990). Avian foraging: theory, methodology, and applications.. Stud Avian Biol.

[36] Cockburn A (1991). An introduction to evolutionary ecology..

[37] Pugesek BH, Diem KL (1983). A multivariate study of the relationship of parental age to reproductive success in California gulls.. Ecol.

[38] Buckley FG, Buckley PA (1974). Comparative feeding ecology of wintering adult and juvenile royal terns (Aves - Laridae - Sterninae).. Ecol.

[39] Dunn EK (1972). Effect of age on fishing ability of sandwich terns Sterna sandvicensis.. Ibis.

[40] Wilson RP, Zimmer I (2004). Inspiration by Magellanic penguin: reduced swimming effort when under pressure.. Mar Ecol Prog Ser.

[41] Wilson RP, Shepard ELC, Gomez-Laich A, Frere E, Quintana F (2010). Pedalling downhill and freewheeling up; a penguin perspective on foraging.. Aqua Biol.

[42] Greig SA, Coulson JC, Monaghan P (1983). Age-related differences in foraging success in the Herring Gull (*Larus argentatus*).. Anim Behav.

[43] Grémillet D, Wilson RP, Storc S, Gary Y (1999). Three-dimensional space utilization by a marine top predator.. Mar Ecol Prog Ser.

[44] Rose MR (1991). Evolutionary biology of aging..

[45] Elliott KH, Davoren GK, Gaston AJ (2008). Time allocation by a deep-diving bird reflects prey type and energy gain.. Anim Behav.

[46] Finch CE (1990). Longevity, senescence, and the genome..

[47] Harman D (1956). Aeging: a theory based on free radical and radiation chemistry.. J Gerontol.

[48] Devevey G, Bruyndonckx N, von Houwald F, Studer-Thiersch A, Christe P (2010). Age-specific vatiation of resistance to oxidative stress in the greater flamingo (*Phoenicopterus ruber roseus*).. J Ornithol.

[49] MacNulty DR, Smith DG, Vucetich JA, Mech LD, Stahler DR (2009). Predatory senescence in ageing wolves.. Ecol Let.

[50] Gurven M, Kaplan H, Gutierrez M (2006). How long does it take to become a proficient hunter? Implications for the evolution of extended development and long life span.. J Human Ecol.

[51] Nisbet I, Apanius V, Friar MS (2002). Breeding performance of very old common terns.. J Field Ornithol.

[52] Bunce A, Ward SI, Norman FI (2005). Are age-related variations in breeding performance greatest when food availability is limited?. J Zool.

